# Whole genome sequence of *Mycobacterium kansasii* isolates of the genotype 1 from Brazilian patients with pulmonary disease demonstrates considerable heterogeneity

**DOI:** 10.1590/0074-02760180085

**Published:** 2018-06-25

**Authors:** Edson Machado, Sidra Ezidio Gonçalves Vasconcellos, Camillo Cerdeira, Lia Lima Gomes, Ricardo Junqueira, Luciana Distasio de Carvalho, Jesus Pais Ramos, Paulo Redner, Carlos Eduardo Dias Campos, Paulo Cesar de Souza Caldas, Ana Paula Chaves Sobral Gomes, Telma Goldenberg, Fatima Fandinho Montes, Fernanda Carvalho de Queiroz Mello, Vinicius de Oliveira Mussi, Elena Lasunskaia, Dick van Soolingen, Antonio Basílio de Miranda, Leen Rigouts, Bouke C de Jong, Conor J Meehan, Marcos Catanho, Philip N Suffys

**Affiliations:** 1 Fundação Oswaldo Cruz-Fiocruz, Instituto Oswaldo Cruz, Laboratório de Genômica Funcional e Bioinformática, Rio de Janeiro, RJ, Brasil; 2 Fundação Oswaldo Cruz-Fiocruz, Instituto Oswaldo Cruz, Laboratório de Biologia Molecular Aplicada a Micobactérias, Rio de Janeiro, RJ, Brasil; 3 Fundação Oswaldo Cruz-Fiocruz, Instituto Oswaldo Cruz, Laboratório de Biologia Computacional e Sistemas, Rio de Janeiro, RJ, Brasil; 4 Fundação Oswaldo Cruz-Fiocruz, Escola Nacional de Saúde Pública, Centro de Referência Professor Hélio Fraga, Laboratório de Referência Nacional para Tuberculose, Rio de Janeiro, RJ, Brasil; 5 Universidade Federal do Rio de Janeiro, Instituto de Doenças do Tórax, Rio de Janeiro, RJ, Brasil; 6 Universidade Estadual do Norte Fluminense Darcy Ribeiro, Laboratório de Biologia do Reconhecer, Campos dos Goytacazes, RJ, Brasil; 7 National Institute for Public Health and the Environment, Bilthoven, the Netherlands; 8 Institute of Tropical Medicine, Unit of Mycobacteriology, Antwerp, Belgium

**Keywords:** Mycobacterium kansasii, genomes, lung disease, genotype I, diversity, drug resistance

## Abstract

*Mycobacterium kansasii* is an opportunistic pathogen and one of the most commonly encountered species in individuals with lung disease. We here report the complete genome sequence of 12 clinical isolates of *M. kansasii* from patients with pulmonary disease in Brazil.

Nontuberculous *Mycobacterium* (NTM) species are widespread in the (man-made) environment and some species cause opportunistic infections in humans. *Mycobacterium kansasii*, frequently isolated from tap water, is a slow-growing photochromogenic NTM and a pathogen that is commonly isolated from patients with pre-existing lung disease, similar to other NTM clinical species such as *M. abscessus*, *M. avium* complex, *M. malmoense* and *M. xenopi*.^1^ In the USA and South America *M. kansasii* is the second most isolated NTM after *M. avium* complex^2^ ^,^ ^3^ while in Rio de Janeiro, it is the most frequent NTM to cause pulmonary disease.^4^ Besides chronic bronchopulmonary disease, *M. kansasii* causes other clinical manifestations such as lymphadenitis^5^, skin and soft tissue infection^6^, tenosynovitis, arthritis, osteomyelitis and disseminated infection in patients co-infected with human immunodeficiency virus.^7^

Seven major genotypes (I to VII) of *M. kansasii* have been described and while human isolates are mainly of types I and II (with type II mainly HIV-related)^8^ ^,^ ^9^, environmental isolates are mostly of the other subtypes.^10^ Type I has been described as a heterogeneous group, incompletely characterized on the genomic level.^11^ ^,^ ^12^ Recently, the genome sequence of the ATCC strain 12478 Hauduroy isolated in Kansas in 1955 has been compared with that of the *M. tuberculosis* strain H37Rv^13^, while other *M. kansasii* genomes are available from environmental^14^, human^15^ and simian^16^ sources.

Here, we report the genome sequence of 12 clinical *M. kansasii* isolates belonging to genotype I, as determined by *hsp*65 sequencing. The strains were isolated from human Brazilian patients with pulmonary disease and possibly earlier tuberculosis. Nine isolates were from sputum, two from bronchoalveolar lavage (7287 and 10742) and one of unknown origin (1580). Isolates were from residents of the state of Rio de Janeiro (n = 7), Pernambuco (n = 3), Rio Grande do Sul (n = 1), and Santa Catarina (n = 1). Genomic DNA libraries were constructed using the Nextera XT DNA library kit and whole-genome shotgun sequencing was performed on the Illumina HiSeq2500 platform, generating paired end reads of 2 × 100 bp. Genomes were *de novo* assembled using SPAdes software version 3.11.1^17^, and annotated with RAST^18^ (Table). No evidence for the presence of plasmids was observed.

**TABLE:** Genome features, predicted genes, and GenBank accession numbers of *Mycobacterium kansasii* strains isolated in Brazil from patients with pulmonary disease

Isolate	Accession	Location	Type (hsp65)	Reads	Genome size (bp)	Contigs	Coverage (x)	Genes
BR3657	PQOL00000000	RJ	1B^d^	18,094,408	6,374,282	294	80.90	5,965t
BR6498^*ª*^	PQOM00000000	RS	1A	9,038,224	6,225,838	317	37.37	5,804
BR7287	PQON00000000	RJ	1A	19,684,890	6,282,326	274	79.59	5,842
BR6884	PQOO00000000	RJ	1A	21,305,254	6,411,468	280	125.91	5,980
BR6849	PQOP00000000	SC	1A	16,399,304	6,464,047	265	78.89	6,023
BR10953^*b*^	PQOQ00000000	RJ	1A	14,895,938	6,284,879	297	52.02	5,850
BR10742^*b*^	PQOR00000000	RJ	1A	14,634,490	6,289,619	282	53.58	5,864
BR1580	PQOS00000000	RJ	1B	20,820,372	6,307,487	226	143.20	5,913
BR4404	PQOT00000000	RJ	1B	4,686,134	6,282,923	466	31.43	5,880
BR8835^*ª*^	PQOU00000000	PE	1B	16,906,236	6,228,381	321	53.45	5,775
BR8837^*c*^	PQOV00000000	PE	1B	13,311,444	6,213,268	247	75.41	5,773
BR8839^*c*^	PQOW00000000	PE	1B	9,434,892	6,178,862	270	52.48	5,727

*a*: resistant to rifampicin; *b* and *c* are pairs of isolates obtained from a single patient each. Isolates were from patients residents of Rio Grande do Sul (RS), Rio de Janeiro (RJ), Santa Catarina (SC) and Pernambuco (PE); *d*: 1B differs from the *hsp*65 sequence 1A of the ATCC 12478 strain by a SNP A to G at genome position 39635339 (GenBank KJ186611.1).

To evaluate the overall similarity of the genomes of the isolates from Brazil with that of the isolate ATCC 12478, we also compared the 12 genomes with the ATCC reference genome using the BLAST Ring Image Generator (BRIG) program^19^ (Fig. 1).

We observed many deletions spread in the genomes that were either shared by all or part of the isolates. Three isolates (1580, 3657 and 4404) presented a region of difference of about 27 kb causing the loss of five helicases, a restriction endonuclease and two hypothetical proteins. We also observed multiple deletions shared by the isolates 1580, 3657, 4404, 8835, 8837 and 8839 (Fig. 1).

**Fig. 1:**
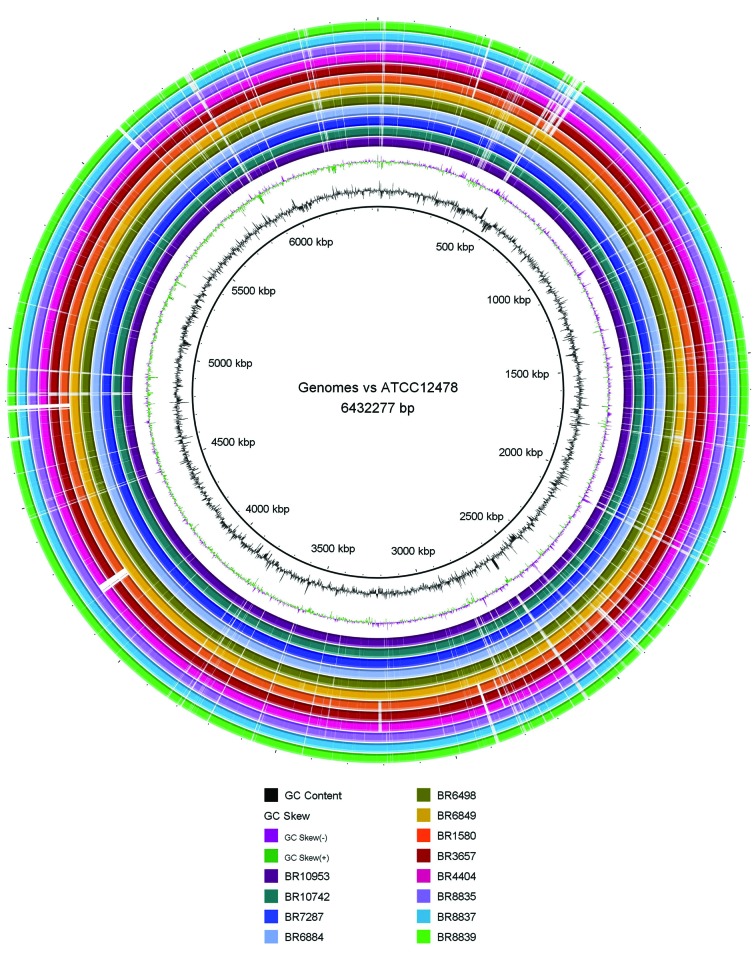
BLAST ring image of the 12 Brazilian genomes against the ATCC 12478 reference genome.

In addition, a reference-based single-nucleotide polymorphism (SNP) calling was performed against the genome of the *M. kansasii* ATCC 12478 strain using both Snippy (version 3.2; https://github.com/tseemann/snippy) and the wgSNP module of BioNumerics v7.6 (Applied Maths, Sint-Martens-Latem, Belgium). Most isolates presented homogeneously distributed SNPs over the entire genome, except for isolates 6849 and 6498 which clearly presented regions of higher SNP frequency (data not shown). As demonstrated by a Neighbour-Joining phylogenetic tree based on 5,607,341 sites and 1000 bootstrap replicates (Fig. 2), three groups were observed when compared to the genome of the ATCC strain, one presenting less than 100 SNP (n = 4) (middle part), a second with either less than 5,000 SNPs (n = 1) and more than 10,000 SNPs (n = 1) (lower part) and a third group with more than 10,000 SNPs (n = 6) (upper part). We observed an association between SNP-based grouping and phylogeny on basis of large deletions, that led us suggest that the third group has evolved from the common ancestor.

From two patients, we performed whole genome sequencing of two isolates each; the pair of isolates from a patient from Rio de Janeiro (10742 and 10953) had 0 SNPs difference; the second pair from a patient from Recife (8837 and 8839) presented 277 mutations (data not shown).

**Fig. 2:**
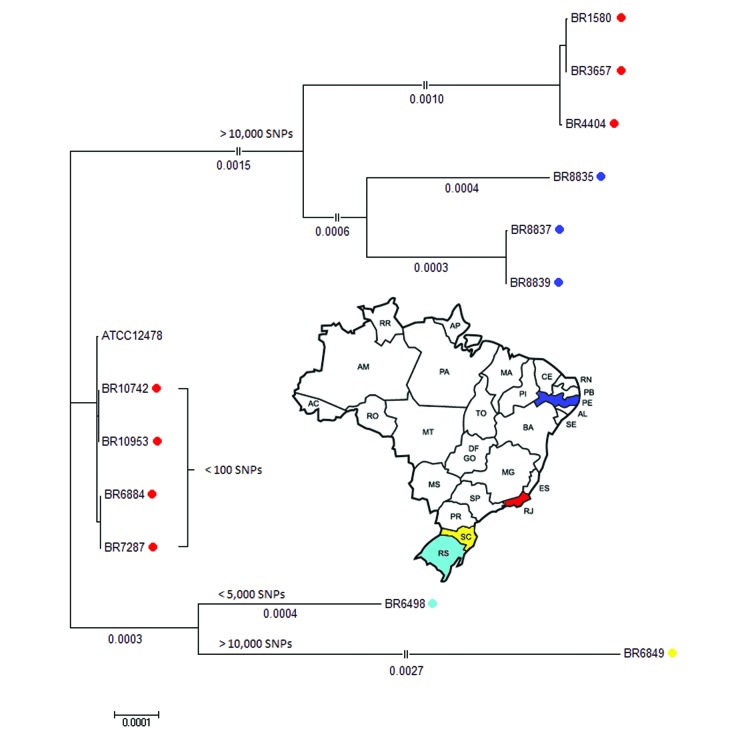
Neighbor-Joining SNP-based tree of the 12 Brazilian isolates and the ATCC 12478 reference genome.

Additionally, two isolates (6498 and 8835) presented a Minimal Inhibitory Concentration of > 1 µg/mL for rifampicin *in vitro* and both presented a non-synonymous SNP, localized respectively at position 4267612 (C to G) and 4267613 (A to C), causing an amino acid change in codon 411 of *rpo*B of respectively Gln to Glu and of Gln to Pro. We as such confirm the scarce existing data on the association between resistance against rifampicin and presence of point mutations in *M. kansasii rpo*B gene. Indeed, point mutations in *rpo*B gene are the main reason for resistance against rifampicin and this has been confirmed in several bacteria. Also, molecular determinants of drug resistance in *M. kansasii* has been recently suggested by Bakuła et al.^20^. Among the present isolates, we observed considerable differences of *in vitro* virulence in macrophages as measured by growth and induction of necrosis and of cytokine production (data not shown).

In conclusion, these are preliminary data on the variability within the *M. kansasii* genotype 1 in Brazil and, even on the basis of this small sample number, we evidenced three genetic groups that are separated by the presence of large number of SNPs throughout the genome and by the pattern of large deletions. One grouping of strains from Rio de Janeiro is highly similar to the ATCC strain Hauduroy (< 100 SNPs) that was isolated more than 70 years ago in the US. A second group of human isolates from Rio de Janeiro and Pernambuco presented over 10,000 SNPs when compared to the ATCC strain. We also observed an association between genotypes and geographic origin of isolates, separating those from the states of Rio de Janeiro, Pernambuco and Santa Catarina. Our observation of considerable differences of *in vitro* and *in vivo* virulence and their differences on the genome level is under investigation.
